# BUILDING H.O.U.S.E. (HEALTHY OUTCOMES USING A SUPPORTIVE ENVIRONMENT) FOR LGBTQ OLDER ADULTS

**DOI:** 10.1093/geroni/igz038.3528

**Published:** 2019-11-08

**Authors:** Amy Rosenwohl-Mack, Matt Beld, Meredith Greene, Karyn Skultety, Leslie Dubbin, Madeline B Deutsch, Jason D Flatt

**Affiliations:** 1 University of California, San Francisco, San Francisco, California, United States; 2 Institute for Health & Aging, School of Nursing, University of California, San Francisco, San Francisco, California, United States; 3 School of Medicine, University of California, San Francisco, San Francisco, California, United States; 4 Openhouse, San Francisco, California, United States; 5 Department of Social & Behavioral Sciences, School of Nursing, University of California, San Francisco, San Francisco, California, United States; 6 Center of Excellence for Transgender Health, University of California, San Francisco, San Francisco, California, United States

## Abstract

Lesbian, gay, bisexual, transgender, and queer (LGBTQ) older adults face unique challenges in finding affordable, inclusive, and supportive housing. These challenges may be due to discrimination, income disparities, and higher rates of health problems compared to cisgender heterosexual seniors. To our knowledge, this is the first longitudinal study of the health and wellbeing of older adults who move into LGBTQ-welcoming, affordable senior housing. Participants completed a brief baseline survey at the time of their housing lottery application. Questions focused on physical, psychological, and social health and current health service use. We calculated descriptive statistics on health status at baseline. 184 participants completed the baseline survey, mean age was 68 years (SD 5.2), and nearly 75% reported an annual income under $30,000. Almost half reported a diagnosis of hypertension, 40% depression, 27% anxiety, and 25% HIV/AIDS. Around 70% reported their health as good to excellent, 21% fair, and 9% poor or very poor. However, 58% reported their physical activities were at least somewhat limited by their physical health, 43% reported difficulties with balance or walking, and 32% reported memory problems. Nearly 3% had been admitted into the hospital and 10% had visited the emergency room in the past 30 days. In terms of social wellbeing, 63% felt isolated from others at least some of the time. In summary, LGBTQ older adults seeking affordable senior housing report relatively good health, although they also experience functional and social difficulties. New forms of housing that are explicitly LGBTQ-welcoming may help address these health challenges.

